# The Timeless (*Tim*) *Gene* Participates in Circadian Rhythm Regulation of the Ridgetail White Prawn, *Exopalaemon carinicauda*

**DOI:** 10.3390/ani16182834

**Published:** 2026-09-09

**Authors:** Caijuan Tian, Jixuan Zhu, Xuanjian Chen, Wanxin Ma, Panpan Niu, Peiyi Bian, Huan Gao, Yuquan Li

**Affiliations:** 1 School of Marine Science and Engineering, Qingdao Agricultural University, Qingdao 266237, China; 2 Jiangsu Key Laboratory of Marine Biotechnology, Jiangsu Ocean University, Lianyungang 222005, China; 3 Co-Innovation Center of Jiangsu Marine Bio-Industry Technology, Lianyungang 222005, China; 4 The Jiangsu Provincial Platform for Conservation and Utilization of Agricultural Germplasm, Nanjing 210014, China

**Keywords:** *Exopalaemon carinicauda*, circadian clock gene, rhythmicity, photoperiod, the ecdysone receptor

## Abstract

We previously found that period (*per*) may exert a positive regulatory effect on ovarian development under the modulation of circadian rhythms. This study further explores the function of the core clock gene *timeless*. Thus, we cloned the *tim* gene (*Ec-tim*) from the ridgetail white prawn, *Exopalaemon carinicauda*. The full-length *Ec-tim* cDNA sequence is 6102 bp in length and encodes a protein of 1543 amino acids with a conserved TIM domain. Quantitative real-time PCR (qPCR) analysis revealed that *Ec-tim* displays prominent eyestalk-specific expression in both adult and sexually mature individuals. Under different light–dark (L:D) cycles, *Ec-tim* exhibited regular oscillations under various L:D ratios within a 24 h period, and even under constant darkness (0 L:24 D) (*p* < 0.05), suggesting that *tim* is an endogenous clock gene. After RNAi-mediated silencing of *Ec-tim*, the transcript levels of key circadian clock genes, including *per* and cryptochrome 1 (*cry1*), were significantly downregulated relative to the control group (*p* < 0.01). In contrast, the mRNA level of the ecdysone receptor gene (*EcR*) was markedly upregulated (*p* < 0.01). These findings suggest that *tim* may regulate the expression of *per* and *cry1* to form a trimeric complex, synergistically modulating circadian rhythms to further affect ovarian development in shrimp. Collectively, this study provides valuable references for further research on circadian regulation and reproductive development in crustaceans.

## Simple Summary

In this study, we cloned the full-length sequence of the timeless gene (*Ec-tim*) from ridgetail white prawn, *Exopalaemon carinicauda*. Bioinformatic analysis predicts that the encoded protein contains a conserved TIM domain, verifying the typical structural characteristics of clock proteins. Quantitative real-time PCR (qPCR) detection showed that *Ec-tim* was specifically highly expressed in the eyestalks with developmental stage-dependent expression patterns. Moreover, its expression exhibited circadian oscillation under different light–dark cycles and constant darkness, suggesting that *Ec-tim* is a key endogenous circadian clock gene. RNA interference (RNAi) experiments indicated that silencing *Ec-tim* significantly downregulated the expression of core clock genes period (*per*) and cryptochrome 1 (*cry1*), but markedly upregulated the ecdysone receptor gene (*EcR*). This regulatory relationship suggests that *Ec-tim* may participate in the modulation of ovarian development via the molting hormone pathway, and the detailed molecular mechanism remains to be further explored.

## 1. Introduction

Circadian rhythms are endogenous biological oscillators driving 24 h diel physiological and behavioral cycles. The timeless (*tim*) gene acts as a core component of this conserved regulatory system. First identified from *Drosophila* mutants exhibiting defective pupal development and disrupted locomotor rhythmicity [1], and subsequently functionally characterized in mammals including *Mus musculus* [2,3]. Beyond being a core clock oscillator, *tim* critically regulates cell cycle progression and embryonic development [4]. Loss of *tim* severely disrupts circadian rhythm homeostasis [5], whereas targeted mutations cause substantial embryonic mortality [6]. Mechanistically, *tim* coordinates periodic behavioral outputs via synergistic molecular crosstalk with the period (*per*) and cryptochrome (*cry*) clock genes [7]. In *Drosophila*, the TIM protein undergoes light-dependent phosphorylation and degradation. Upon heterodimerization with PER, TIM promotes nuclear translocation of the protein complex to repress CLOCK/CYCLE transcriptional activity, maintaining the classic autoregulatory feedback loop [8,9,10,11,12]. In other invertebrates such as *Bombyx mori*, TIM forms a trimeric PER–TIM–CRY1 complex to modulate transcription of clock-controlled downstream genes [13].

As a critical environmental parameter, light, including light spectra and photoperiod, regulates multiple physiological processes in crustaceans. Photoreceptors perceive light signals to synchronize endogenous circadian clock machinery, which controls temporal transcription of core clock genes. Light modulates circadian rhythms, growth, molting, metabolism and gonadal development [14]. Alterations in photoperiod affect the input pathways, underlying molecular mechanisms, oscillation amplitudes, phase shifts and entrainment of the circadian system [15]. Disrupted light conditions disturb clock gene rhythmicity and impair decapod reproduction [16]. Reproductive performance relies on the secretion of rhythmic gonadotropins and steroid hormones [17]. Accumulating evidence indicates that this endocrine rhythmicity is controlled by ovarian clock gene expression [18]. Multiple studies have revealed molecular links between circadian timing and reproductive physiology across mammals, birds and insects [19,20]. Functional assays show that silencing ovarian clock genes impairs oocyte ovulation [21], and BMAL1/CLOCK heterodimers temporally coordinate steroid biosynthesis [22]. In crustaceans, seasonal oscillatory expression of *per* and *tim* exhibits correlations with female gonadal development [23]. In the red swamp crayfish *Procambarus clarkii*, light-entrained circadian rhythms regulate ovarian maturation through extraretinal photoreceptors [24]. Crustacean ovarian maturation depends on ecdysteroid endocrine signaling [25]. The ecdysone receptor (*EcR*) is the core receptor of the pathway, playing an important role in promoting ovarian maturation [26]. Nevertheless, whether *tim* is potentially involved in reproductive regulation through the molting hormone pathway remains largely unclear.

In decapod crustaceans, the eyestalk and attached optic ganglia serve as the primary hub for neuroendocrine integration and behavioral rhythmicity [27]. The X-organ/sinus gland (XO-SG) complex synthesizes and secretes key neurohormones, such as crustacean hyperglycemic hormone (CHH) and molt-inhibiting hormone (MIH), which collectively regulate metabolism, development, and reproduction [28]. *Exopalaemon carinicauda* is an economically important native coastal shrimp in China with high nutritional value. Despite its great aquaculture potential, large-scale artificial breeding remains unavailable [29,30,31]. Our previous work identified and functionally characterized the *per* gene in *E. carinicauda* [32]. The present study aims to characterize the circadian function of the *tim* gene and explore its potential crosstalk with the ecdysteroid signaling pathway. This work provides clues between endogenous clock genes and reproductive regulation in crustaceans.

## 2. Materials and Methods

### 2.1. Experimental Materials and Sample Collection

All animals in the study (body weight of 2.06 ± 0.59 g) are from our self-propagating family in our laboratory. Prior to the experiments, shrimp were temporarily placed in a tank (50 cm (Length) × 40 cm (Width) × 30 cm (Height)) maintained at 25 °C, salinity 26 g/L, and pH 8.0 with continuous aeration (dissolved oxygen was maintained above 6.5 mg/L). To stabilize endogenous circadian rhythms, all individuals were acclimated to a 12 h light:12 h dark (12 L:12 D, ZT0–ZT12; light phase: 08:00–20:00) cycle for one week. ZT0 was uniformly defined as 08:00 daily.

We analyzed the expression of the *tim* gene at different developmental stages: fertilized eggs, zoea larvae I–III, juveniles, and post-larvae. From each stage, we collected 20–30 individuals from three independent spawns, obtaining samples from three biological replicates (*n* = 3). Additionally, we analyzed the tissue expression in the eyestalk, gill, hepatopancreas, heart, stomach, intestine, muscle, and ventral nerve from nine adult prawns. We pooled each tissue from three individuals, obtaining in this way three biological replicates (*n* = 3). Finally, we analyzed ovarian expression across five stages of maturation: proliferation (G1), small growth (G2), large growth (G3), maturation (G4), and postpartum recovery (G5) [33]. In each maturation stage, we collected ovarian tissue from three adult females (*n* = 3).

### 2.2. Total RNA Extraction and cDNA First-Strand Synthesis

Total RNA was extracted using the UNIQ-10 column Trizol kit (Sangon, Shanghai, China) following the standard manufacturer’s protocol. RNA purity was determined using NanoDrop spectrophotometry (Quawell Technology, Inc., San Jose, CA, USA) (A260/A280: 1.8–2.2 and A260/A230: ≥1.8). RNA integrity was validated on 1% agarose gels showing conspicuous 28S and 18S rRNA bands. DNase I (TaKaRa) was used to remove any remaining genomic DNA in the samples. Then, 1 μg of total RNA was reverse-transcribed into cDNA using the PrimeScript ™ RT Master Mix kit (TaKaRa, Nanjing, China). The cDNA templates were diluted to 50 ng/μL and stored at −20 °C for qRT-PCR.

### 2.3. Cloning and Biological Information Analysis of Ec-Tim

Gene-specific primers were as illustrated in Table 1. The full-length cDNA of *Ec-tim* was obtained via 5′ and 3′-RACE (Rapid Amplification of cDNA Ends) using the SMARTer RACE 5′/3′ kit (TaKaRa) according to the instruction manual. The open reading frame (ORF) and its corresponding amino acid sequence were identified (http://www.ncbi.nlm.nih.gov/gorf/gorf.html accessed on 1 July 2026). The theoretical isoelectric point and molecular weight of TIM were calculated using DNA Star (V17.5). A phylogenetic tree was reconstructed using the Neighbor-Joining (NJ) method with MEGA 11. Branch support was evaluated through 1000 bootstrap replicates to ensure the reliability of the evolutionary relationships [34].

### 2.4. Experiments on Different Light Spectra and Photoperiods

The experimental design of different spectra and light periods has been elaborated in our previous research [32]. Two independent experiments were conducted in this work. For the light spectrum experiment, 180 individuals were reared under constant white, blue, or red light (15 W, 1500 lx). For the photoperiod experiment, 200 individuals were exposed to five light–dark (L–D) cycles: constant darkness (0 L:24 D), 8 L:16 D (ZT0–ZT8; light phase: 08:00–16:00), 12 L:12 D (ZT0–ZT12; light phase: 08:00–20:00), 16 L:8 D (ZT0–ZT16; light phase: 08:00–24:00), and constant light (24 L:0 D, ZT0–ZT24; light phase: 08:00–08:00 the next day). Eyestalk samples were harvested at 0 (control), 3, 6, 9, 12, 15, 18, 21, and 24 h (ZT0–ZT24) post-treatment across both experiments. Three parallel culture groups were established per treatment, and six biological replicates were harvested at each time point.

### 2.5. RNA Interference

Specific siRNA primers (see Table 1) were designed via the Thermo Fisher online tool (http://rnaidesigner.thermofisher.com/rnaiexpress/design.do accessed on 9 December 2025) and synthesized using the T7 RNAi Transcription Kit (Vazyme, Nanjing, China). The detailed experimental design of RNA interference can be found in our previously published research [32]. A total of 200 individuals were randomly assigned to control and interference groups with three biological replicates per group. Individuals in the interference group were injected with siRNA at a dose of 4 µg/g body weight, whereas the control group received an equivalent volume of normal saline via pericardial cavity injection. Eyestalk tissues were collected at 4, 8, 12, 16, 20, and 24 h (ZT4–ZT24) post-injection, and ovarian tissues were harvested at 12 (ZT12) h and 24 h (ZT24) post-injection. Each time point was sampled with three independent biological replicates.

### 2.6. The mRNA Overexpression of Ec-Tim

The *Ec-tim* coding sequence was cloned into the pSPT18 vector system (Vazyme, Nanjing, China). Primers containing *Bam*HI and *Eco*RI restriction sites and protective bases were used for PCR amplification (Table 1). The resulting products were inserted into the linearized pSPT18 plasmid via homologous recombination using the ClonExpress^®^ II One Step Cloning Kit (Vazyme). The recombinant constructs were then transformed into competent Escherichia coli cells (Sangon), followed by screening and sequence verification. The recombinant plasmid was linearized to serve as a template for in vitro mRNA synthesis using the Easy Cap T7 Co-transcription Kit (Vazyme). A detailed protocol for the overexpression experiment was previously described [32]. For sampling details, refer to Section 2.5. Individuals in the control and overexpression groups were injected with mRNA of *tim* at a dose of 4 µg/g body weight.

### 2.7. qRT-PCR

The qPCR primers are listed in Table 1. qRT-PCR was performed using the SYBR Premix Ex Taq II kit (TaKaRa) on a StepOnePlus Real-Time PCR System (Applied Biosystems, Foster, CA, USA). For this, 18S rRNA served as the internal reference gene. Relative mRNA expression levels were calculated using the 2^−ΔΔCt^ method [35].

### 2.8. Statistical Analysis

All data are shown as mean ± standard deviation (SD) (*n* ≥ 3 biological replicates). Statistical analyses were performed with SPSS 21.0. Shapiro–Wilk and Levene’s tests were used to verify normality and homogeneity before parametric analysis. Student’s *t*-tests were used for two-group comparisons. For three or more groups satisfying parametric assumptions, one-way ANOVA coupled with Bonferroni adjustment was followed by Duncan’s multiple range test for post hoc comparisons. Figures were plotted via GraphPad Prism 10. Significance levels were as follows: * *p* < 0.05, ** *p* < 0.01, *** *p* < 0.001.

## 3. Results

### 3.1. Sequence Characteristics and Homology Analysis of Ec-Tim

**Figure 1:**
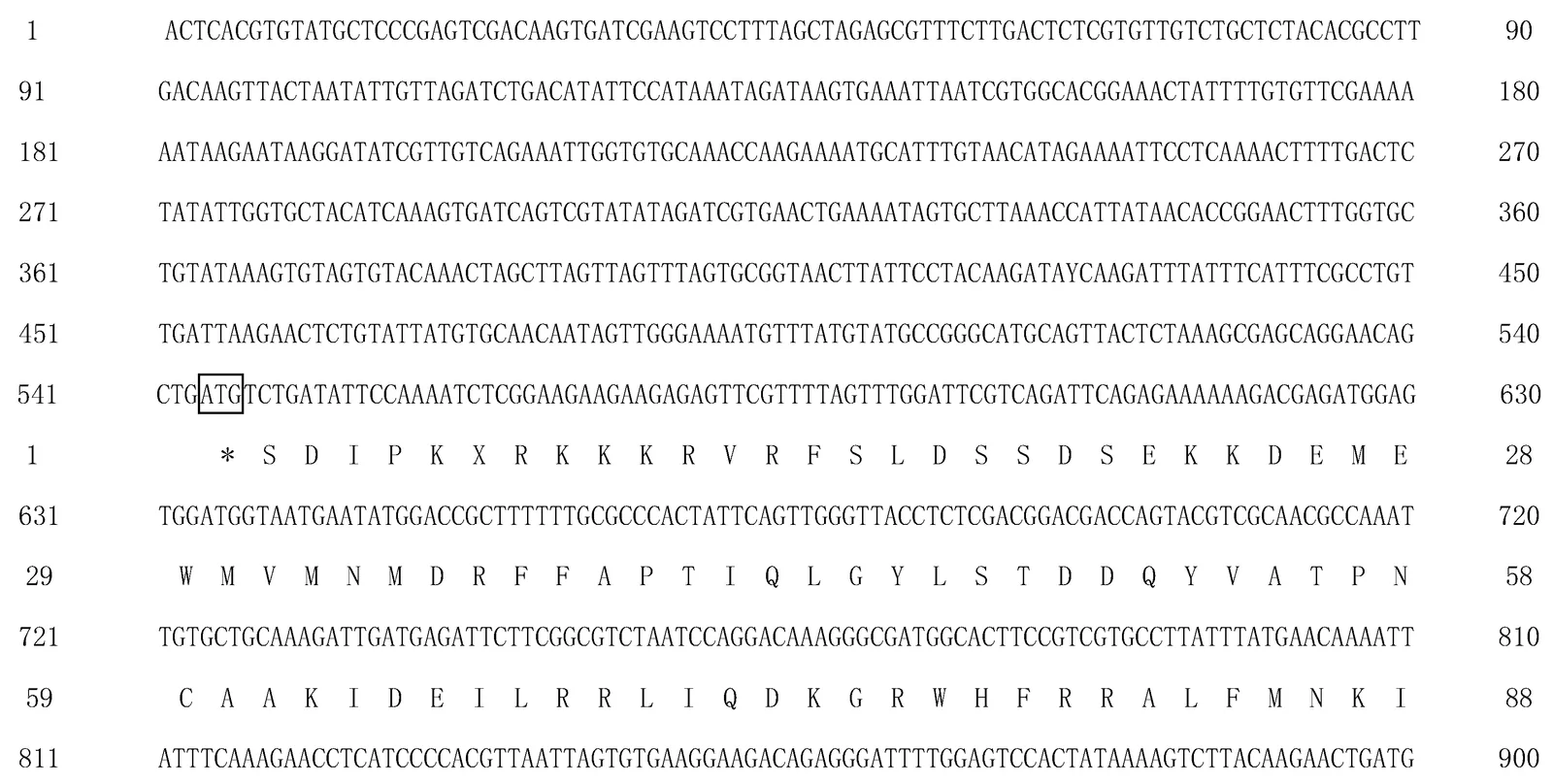

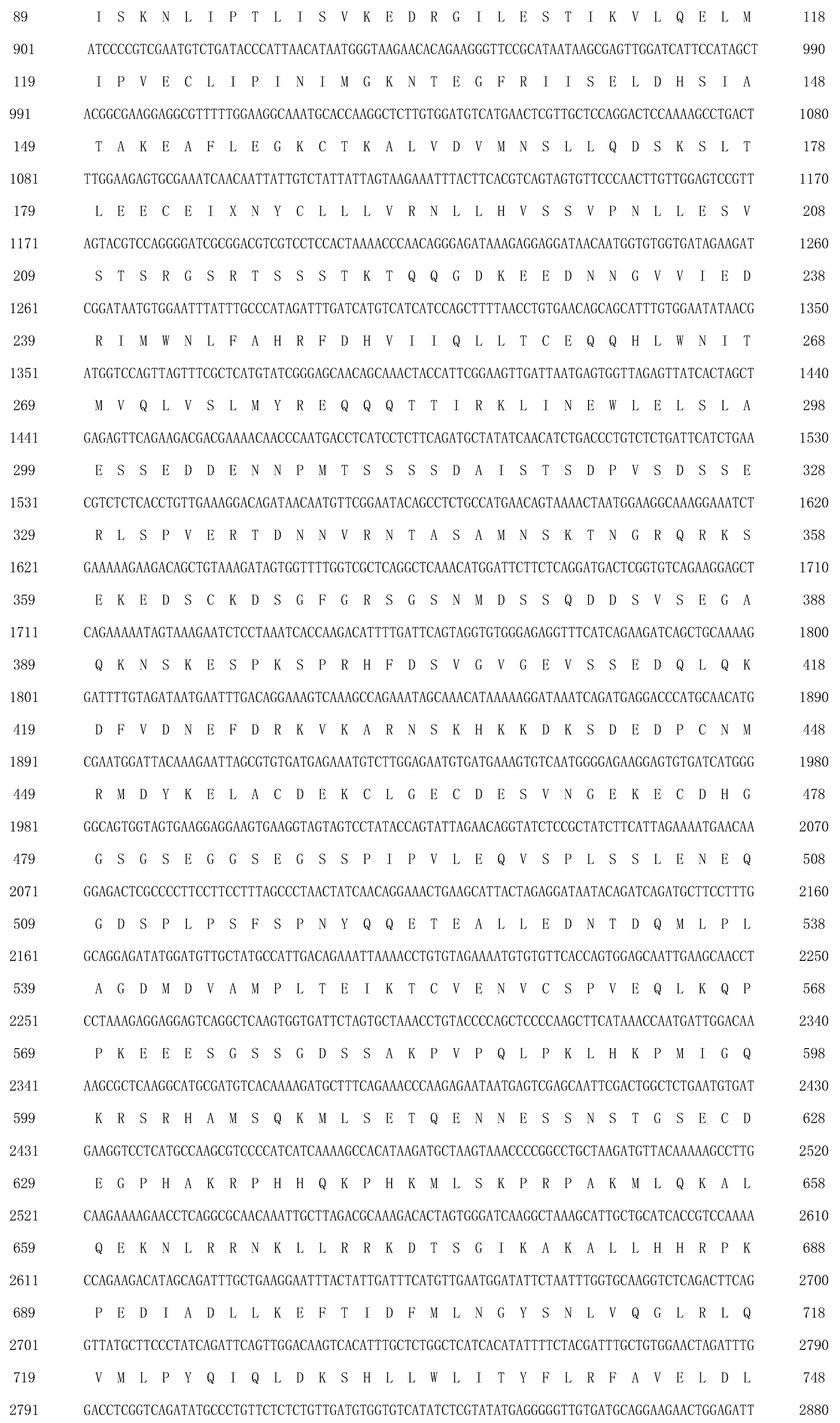

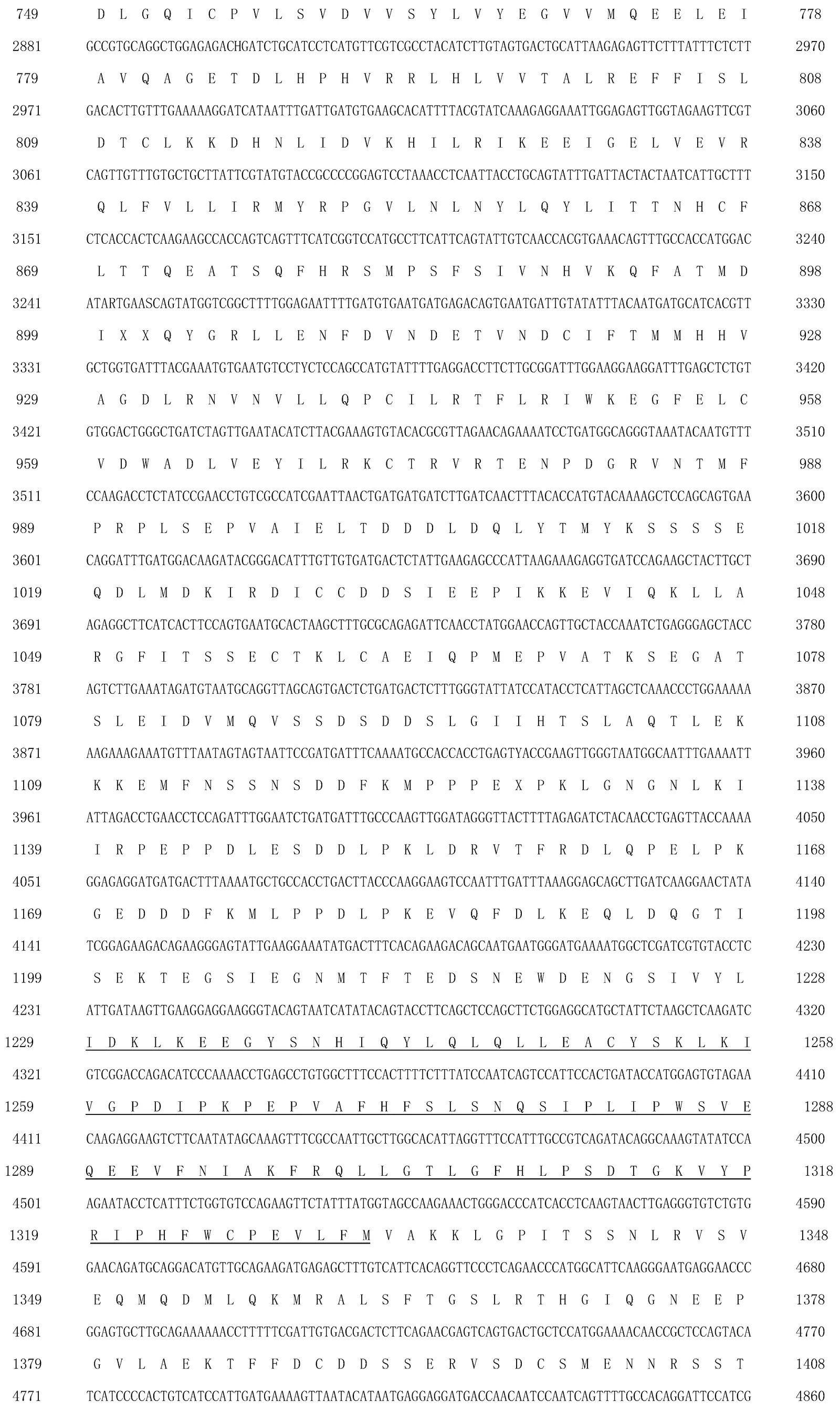

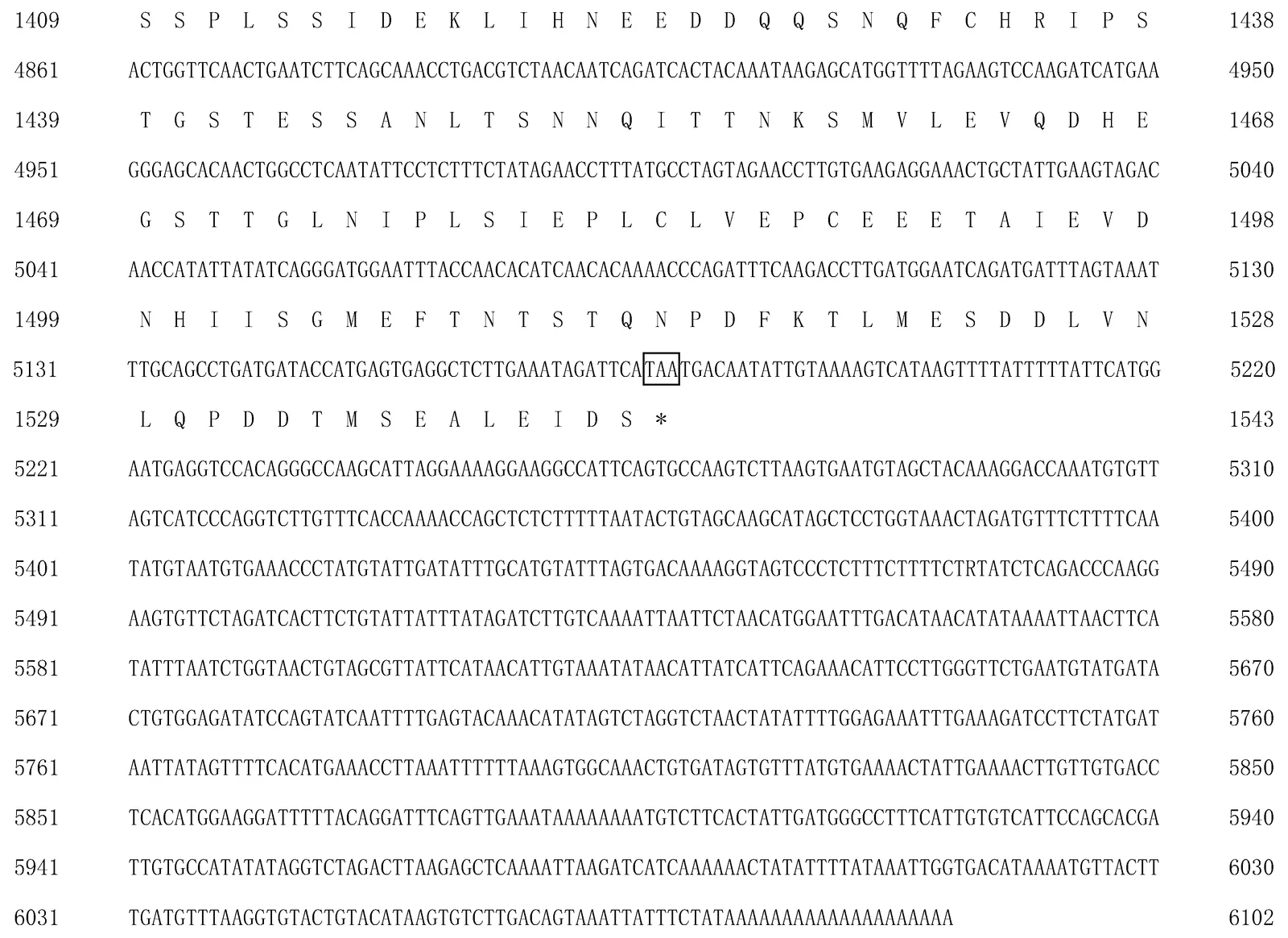
cDNA sequence and amino acid sequence of *Ec-tim*. The start codon (ATG) and the stop codon (TAA) are indicated in the box. * indicates the termination of translation. The underlined part is the TIM domain.

**Figure 2:**
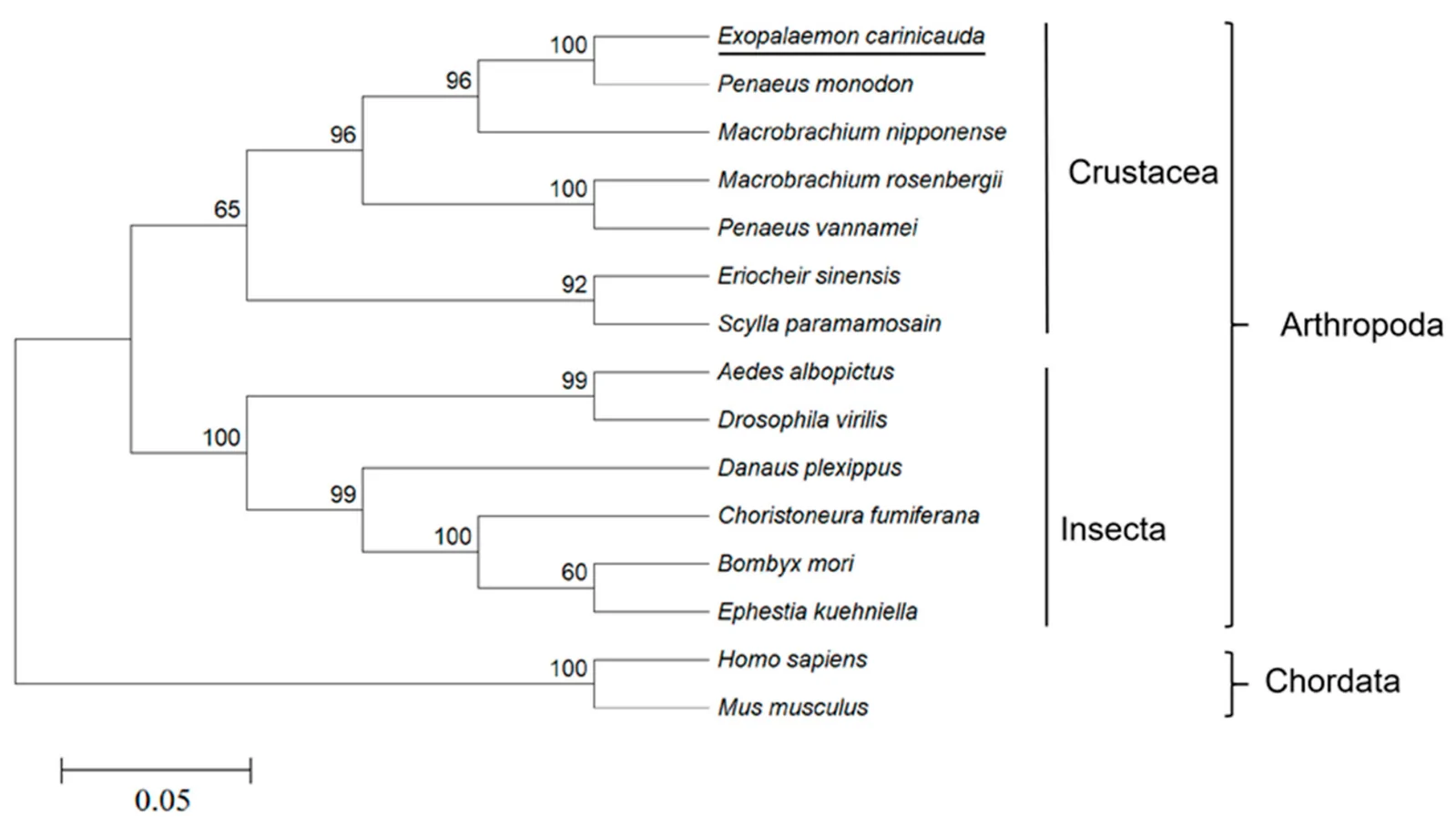
The phylogenetic tree of the TIM amino acid sequence with other species. Note: The horizontal line in the figure is *E. carinicauda.* GenBank accession numbers for TIM: *Penaeus monodon* (XP_037802526.1), *Macrobrachium nipponense* (ANN13871.1), *Macrobrachium rosenbergii* (XP_066947946.1), *Penaeus vannamei* (AYE56134.1), *Eriocheir sinensis* (XP_050729605.1), *Scylla paramamosain* (XP_063887931.1), *Aedes albopictus* (AEX14536.1), *Drosophila virilis* (KRF81798.1), *Danaus plexippus* (OWR44899.1), *Choristoneura fumiferana* (XP_073941711.1), *Bombyx mori* (BBD20567.1), *Ephestia kuehniella* (AJG06482.1), *Homo sapiens* (NP_003911.2), and *Mus musculus* (NP_001157553.1). The number represents the bootstrap values, calculated by MEGA 11 software. A scale of 0.05 represents evolutionary distance.

The *tim* gene sequence has been uploaded to the NCBI database (GenBank: PX656962). The full length of *Ec-tim* cDNA is 6102 bp, consisting of a 543 bp 5′-untranslated region, a 924 bp 3′-untranslated region, and a 4632 bp open reading frame (ORF) encoding 1543 bp amino acids. The predicted molecular weight of the encoded protein is 504.4 kDa, with a theoretical isoelectric point of 6.45. The Ec-tim protein contains a conserved TIM domain at 1229–1331 aa (underlined in Figure 1). Phylogenetic analysis further revealed that Ec-tim has the closest phylogenetic relationship with *Penaeus monodon* (see Figure 2).

### 3.2. Expression of the Tim Gene in Different Developmental Stages and Tissues

**Figure 3:**
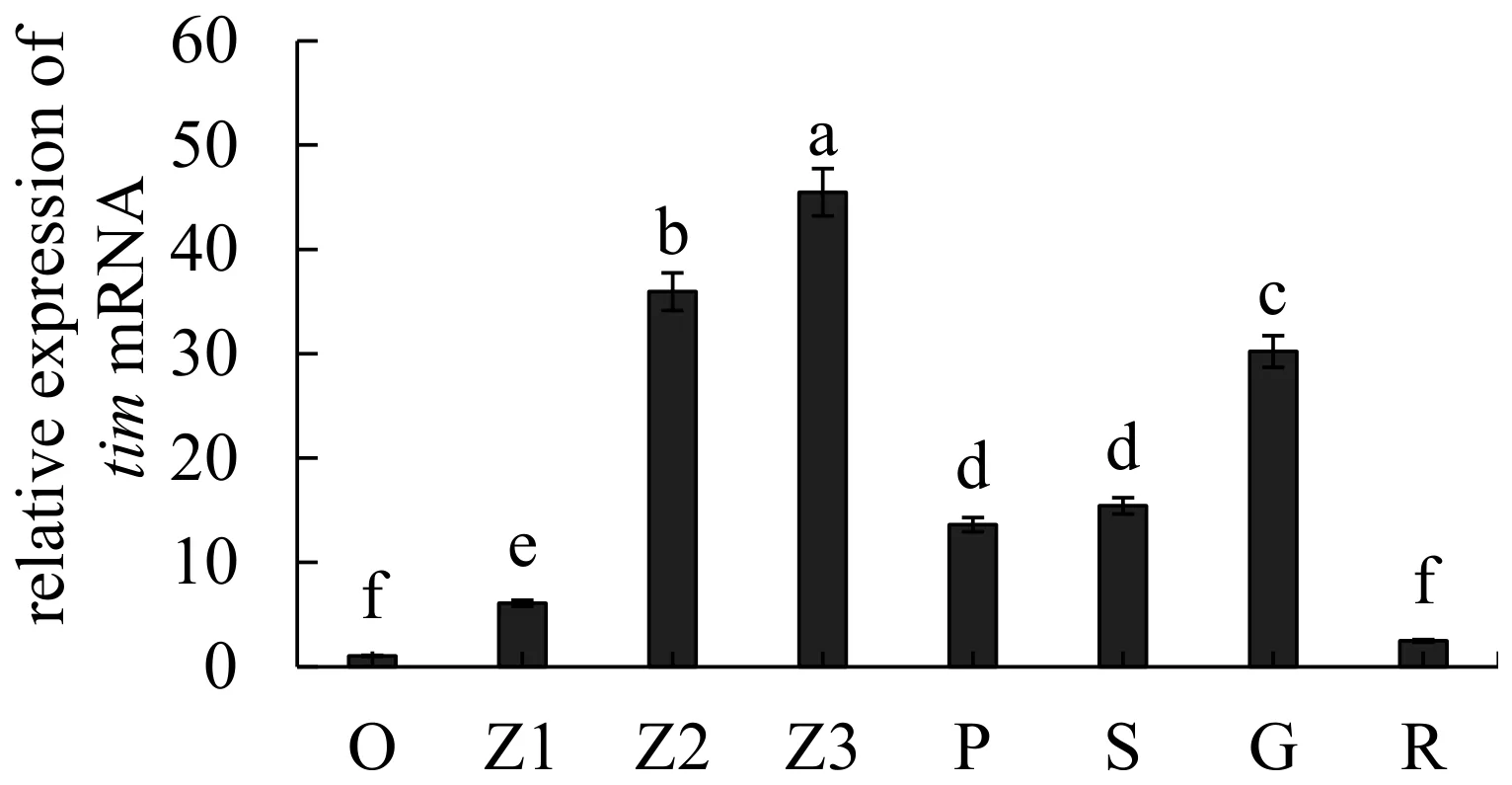
Expression characteristics of the *tim* gene at different development stages of *E. carinicauda*. O, fertilized egg; Z1, Zoea I; Z2, Zoea II; Z3, Zoea III; P, postlarval stage; S, adult shrimp stage; G, gonadal maturity stage; R, postpartum recovery stage. All data are presented as mean ± SD (*n* ≥ 3). Statistical analysis was performed using ANOVA followed by Duncan’s test. Significant differences are presented by different letters (*p* < 0.05).

As shown in Figure 3, *Ec-tim* mRNA levels exhibited a significant upregulation from the fertilized egg stage (the control group) to juvenile development, peaking at the larval stage III (*p* < 0.05). Its relative transcript abundance also increased markedly, and dropped to the minimum level after ovulation (*p* < 0.05).

**Figure 4:**
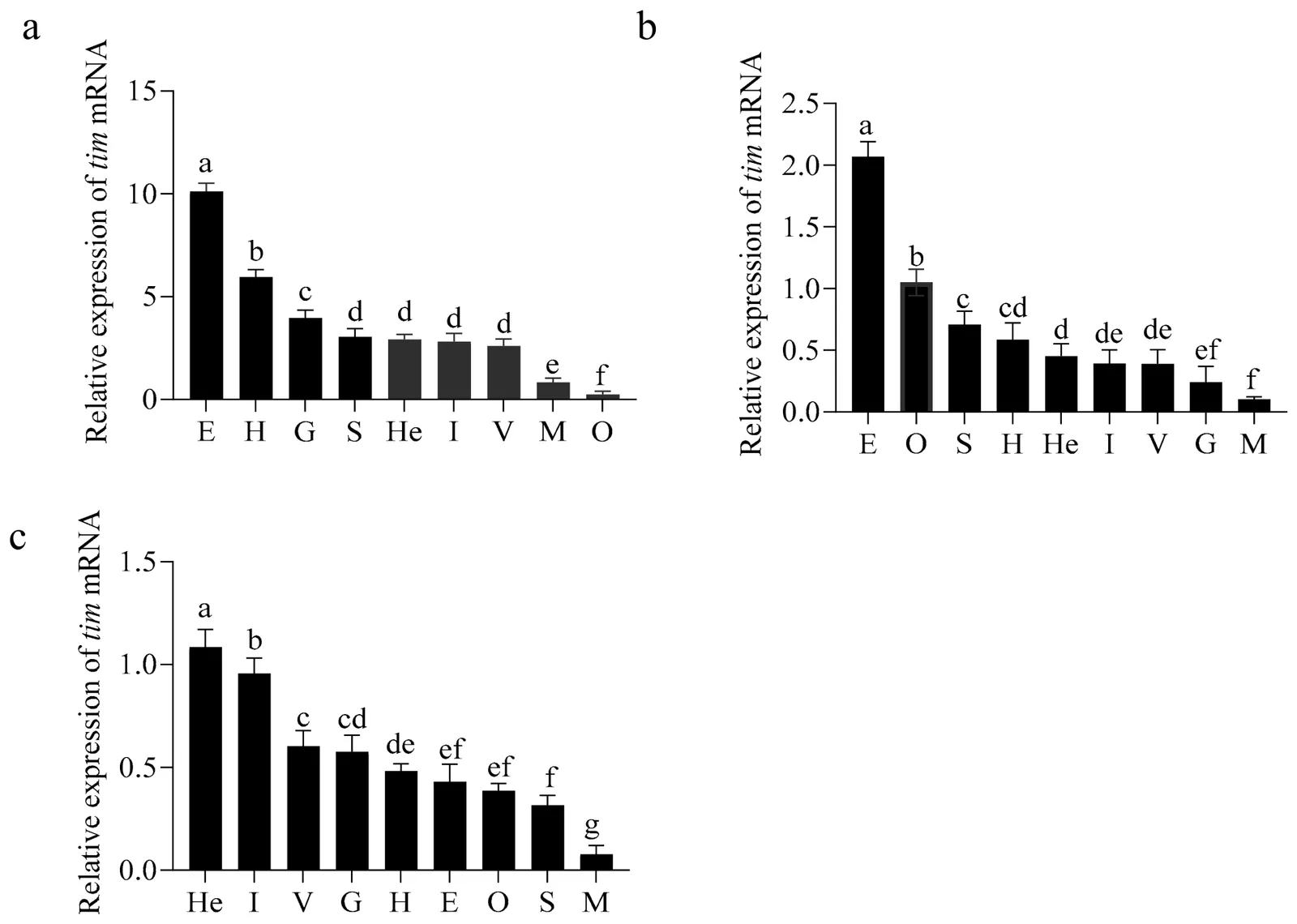
Expression characteristics of the *tim* gene in the tested tissues of *E. carinicauda* at different development stages. (**a**): Adult shrimp stage, (**b**): gonadal maturity stage, (**c**): postpartum recovery period. E, eyestalk; H, hepatopancreas; G, gill; HE, heart; S, stomach; I, intestine; V, ventral nerve; M, muscle; O, gonad. All data are presented as mean ± SD (*n* ≥ 3). Statistical analysis was performed using ANOVA followed by Duncan’s test. Different lowercase letters among the nine tissues present significant differences (*p* < 0.05).

qPCR analysis revealed that the *tim* transcripts were widely distributed in the tested tissues, including the eyestalk, hepatopancreas, gill, stomach, and ovary (as depicted in Figure 4), with stage-specific expression profiles across developmental phases. Relative to the control group (muscle tissue), eyestalk tissue had the highest *Ec-tim* mRNA abundance in adults (Figure 4a), followed by the hepatopancreas and gills (*p* < 0.05), whereas the ovary had the lowest expression (*p* < 0.05). During gonadal maturation (Figure 4b), *tim* mRNA levels were highest in the eyestalk, followed by the ovary and stomach (*p* < 0.05), and muscle tissue exhibited the lowest expression. During the postpartum recovery period (Figure 4c), the *tim* gene was predominantly expressed in the heart, intestine, and ventral nerve, and muscle tissue still possessed the minimum gene expression level (*p* < 0.05).

### 3.3. Expression Profile of Tim in Different Ovary Developmental Stages of E. carinicauda

**Figure 5:**
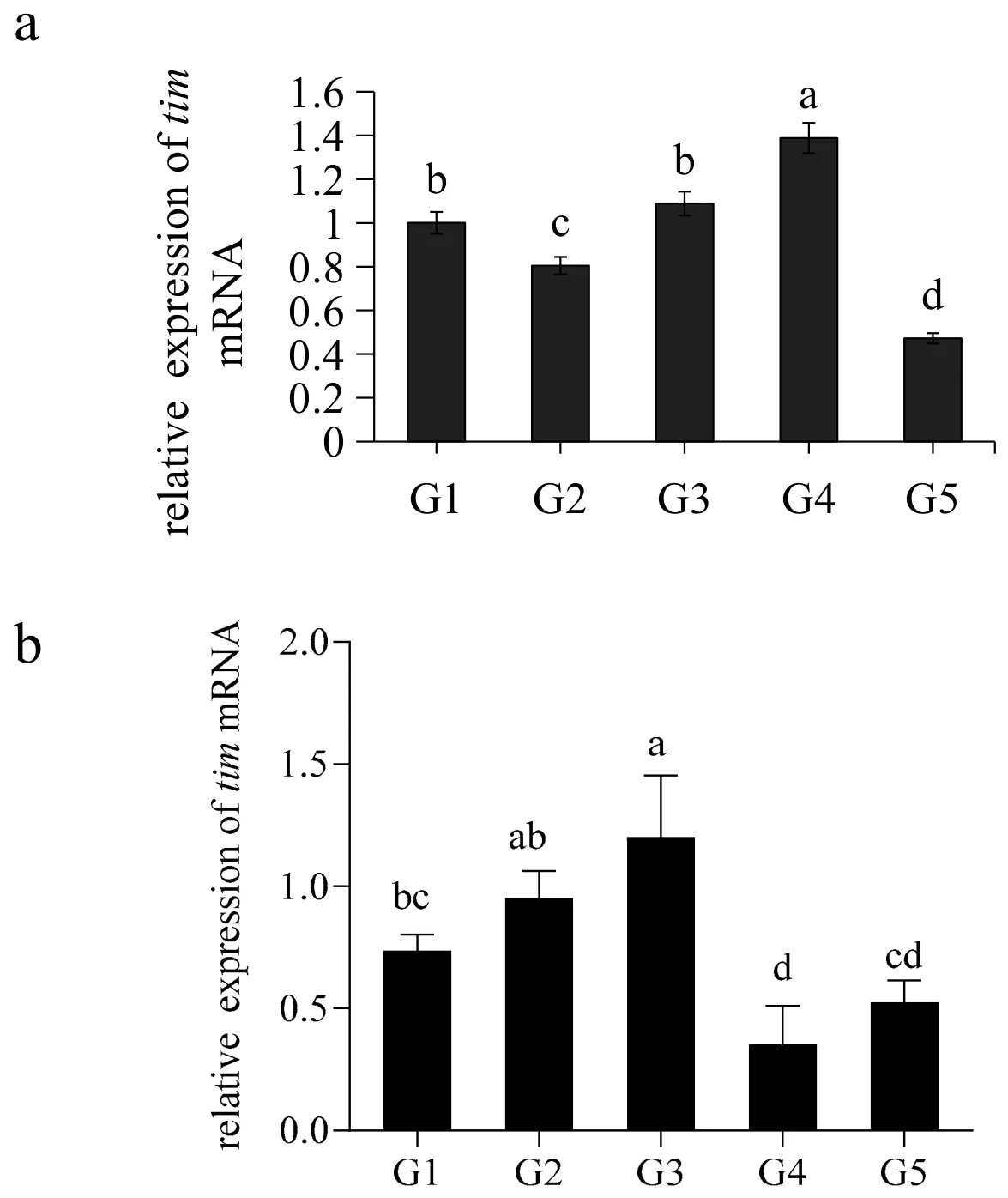
Expression characteristics of the *tim* gene in *E. carinicauda* at different stages of ovary development. (**a**): ovary tissue; (**b**): eyestalk tissue. G1: proliferation period; G2: small growth period; G3: great growth period; G4: mature period; G5: postpartum recovery period. All data are presented as mean ± SD (*n* ≥ 3). Statistical analysis was performed using ANOVA followed by Duncan’s test. Different lowercase letters among different ovary development stages represent significant differences (*p* < 0.05).

As shown in Figure 5a, *tim* transcripts were detectable throughout all five ovarian developmental stages (G1–G5). Transcript levels of the *tim* gene were relatively high at stage G1 (control), and increased gradually during stages G2 and G4 with significant differences (*p* < 0.05). Subsequently, *tim* expression sharply declined to the lowest level across all groups at stage G5. Meanwhile, in eyestalk tissue (Figure 5b), *tim* transcript abundance reached its maximum during the G3 stage and declined to the lowest level at the G4 stage.

### 3.4. The Tim Gene Expression After Exposure to Different Light Spectra and Photoperiods

**Figure 6:**
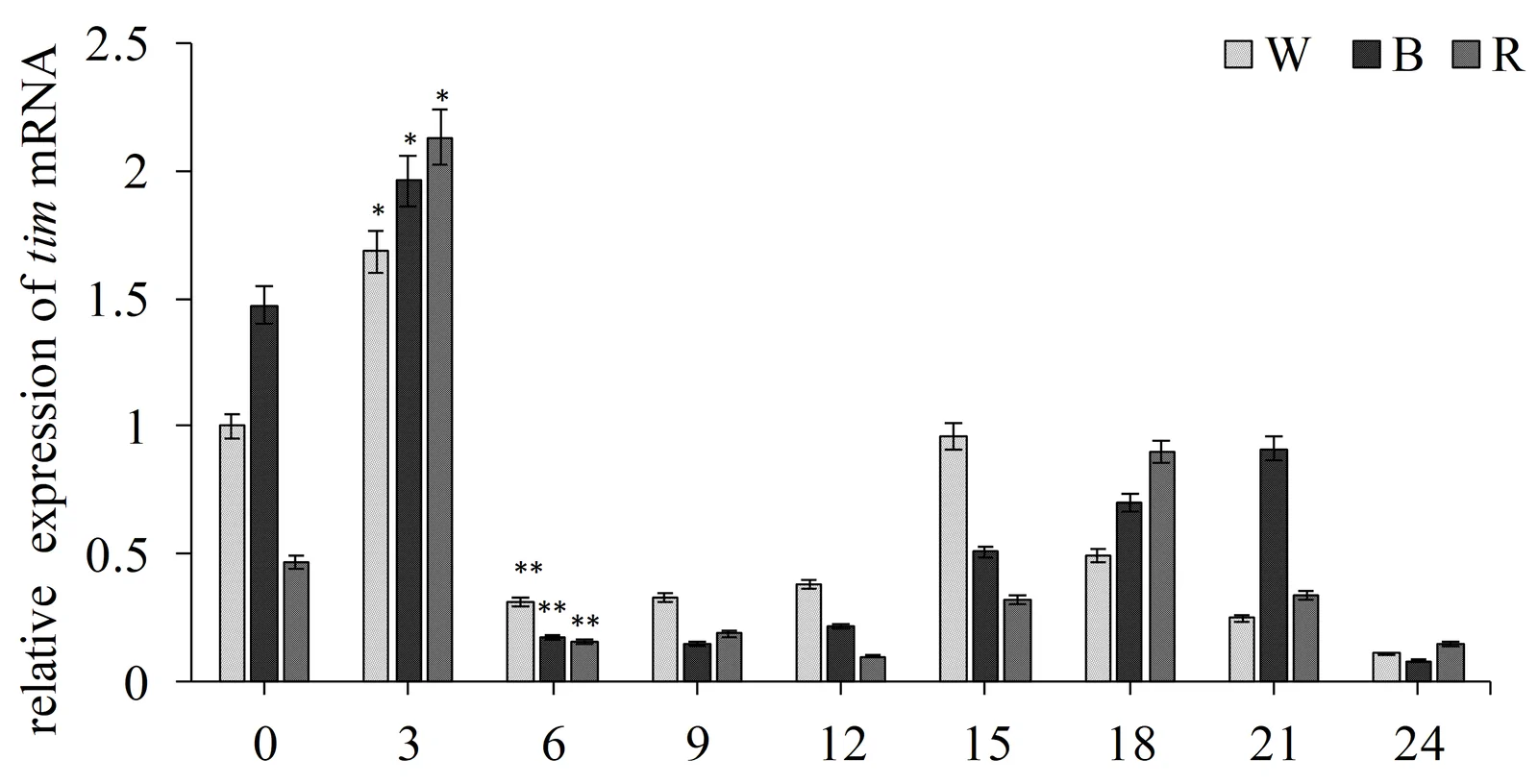
Expression characteristics of the *tim* gene under different light colors at 0 h, 3 h, 6 h, 9 h, 12 h, 15 h, 18 h, 21 h and 24 h. W: white light; B: blue light; R: red light. All data are presented as mean ± SD (*n* ≥ 6). All statistical analysis was performed using ANOVA followed by Duncan’s test. * in 3 h denotes a significant difference compared to the white light group (0 h) (*p* < 0.05). ** in 6 h represents a significant difference compared with the 3 h group (*p* < 0.05).

Following 24 h exposure to white, blue, and red light spectra, *tim* gene expression was detectable in the eyestalk tissue across all three light treatments (as shown in Figure 6). Relative to the control group (0 h), *tim* transcript abundance increased significantly at 3 h post-exposure (*p* < 0.05), followed by a marked reduction at 6 h (*p* < 0.05), rose gradually at 21 h, and reached its lowest level at 24 h. Under continuous white light, *tim* expression peaked at 15 h, whereas for blue and red lights, the expression peaked at 21 h and 18 h, respectively.

**Figure 7:**
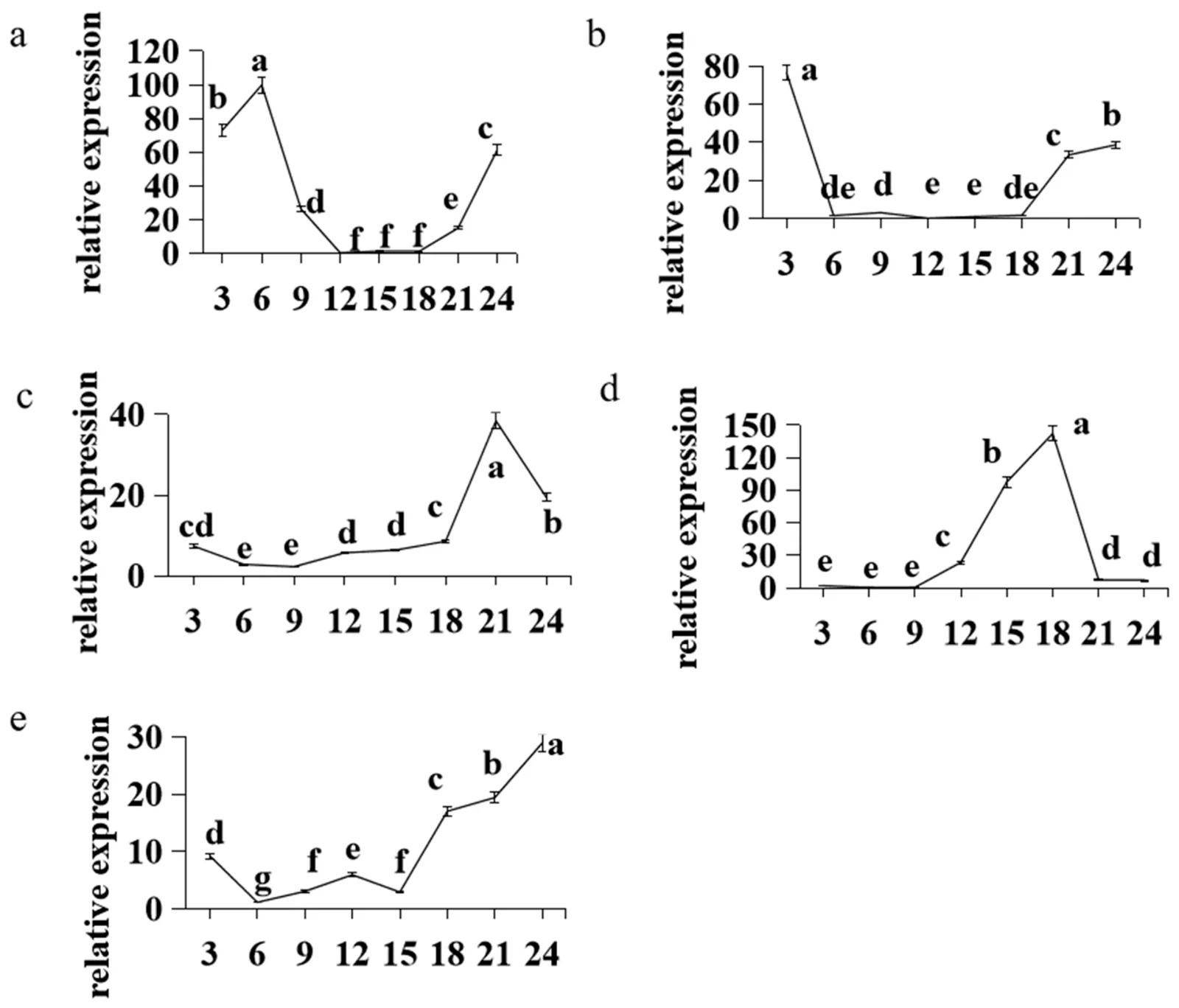
Expression characteristics of the *tim* gene in *E. carinicauda* at 3 h, 6 h, 9 h, 12 h, 15 h, 18 h, 21 h, and 24 h under different photoperiods. (**a**) 0 L:24 D; (**b**) 8 L:16 D; (**c**) 12 L:12 D; (**d**) 16 L:8 D; (**e**) 24 L:0 D. All data are presented as mean ± SD (*n* ≥ 6). Statistical analysis was performed using ANOVA followed by Duncan’s test. Different lowercase letters among different time points present significant differences (*p* < 0.05).

Further exploring the response of *tim* to different photoperiods, the results demonstrated that photoperiod changes altered the phase, amplitude, and expression level of the *tim* gene rhythmic expression profile (Figure 7). The *tim* gene exhibited regular oscillations with alternating rise and fall under 0 L:24 D (Figure 7a). Under 24 L:0 D (control, Figure 7e), *tim* expression fluctuated continuously, increased gradually, and peaked at 24 h. Under 8 L:16 D, 12 L:12 D, and 24 L:0 D, although the oscillation rhythm of *tim* was similar to that under 0 L:24 D, the amplitude decreased and shifted in phase backward by 3–6 h (Figure 7b,c). Under a 16 L:8 D photoperiod (Figure 7d), the amplitude of *tim* increased, and reached its peak at 18 h.

### 3.5. RNA Interference and mRNA Overexpression of the Tim Gene

**Figure 8:**
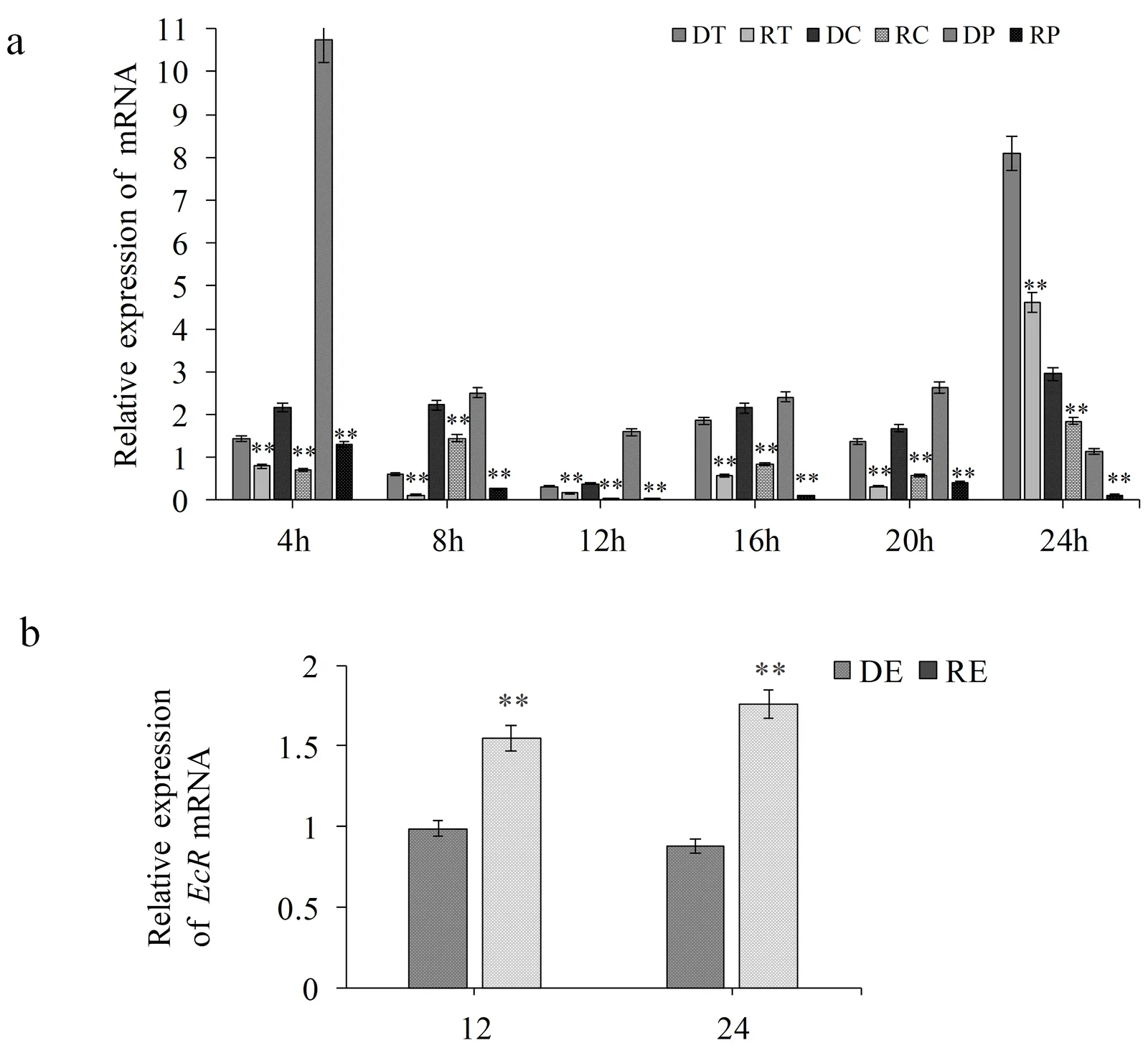
Expression profile of circadian clock genes after *tim* interference. a: Expression profile of *tim*, *per*, and *cry1* in the control (DT, DC, and DP) and interference groups (RT, RC, and RP) in the eyestalk at 4 h, 8 h, 12 h, 16 h, 20 h, and 24 h. b: Expression profile of *EcR* in the control (DE) and overexpression groups (RE) in the ovary at 12 h and 24 h. Data are presented as mean ± SD (*n* ≥ 3). Statistical analysis was performed using ANOVA followed by Duncan’s test (**a**). Student’s *t*-test was also conducted (**b**). Significant differences between the two groups are indicated by ** (*p* < 0.01).

After interfering with *tim*, the mRNA levels of *tim* and other circadian genes, including *per* and *cry1*, were significantly lower in the interference group than in the control group (DT, DC and DP) at all tested time points (*p* < 0.01). The interference efficiency exceeded 50%, indicating successful gene silencing (Figure 8a). Compared with the control group (DE), transcript levels of the ovarian development-related gene *EcR* were significantly upregulated at 12 and 24 h (*p* < 0.01) (Figure 8b).

**Figure 9:**
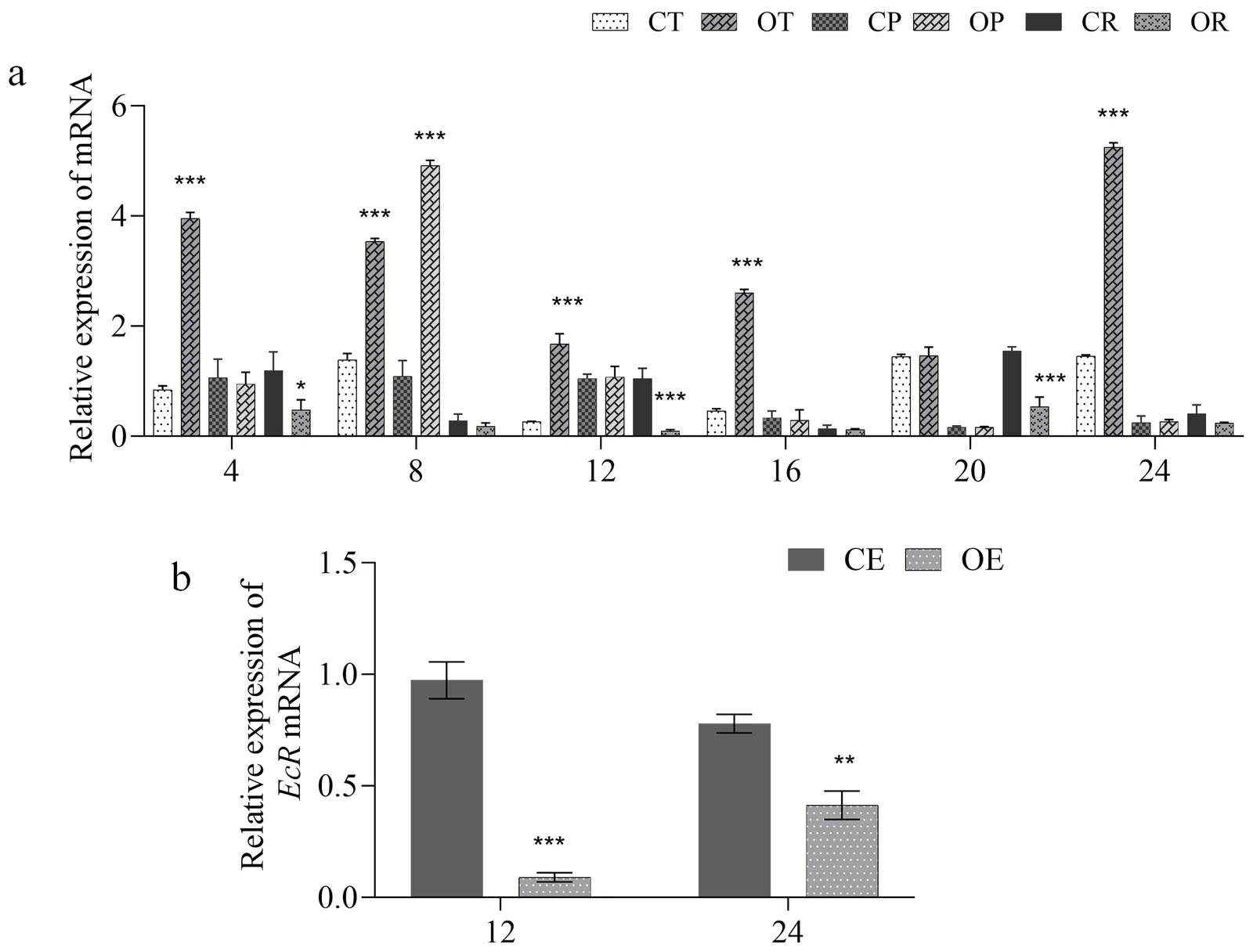
Expression profile of circadian clock genes after tim mRNA overexpression. a: Expression profile of *tim*, *per*, and *cry1* in the control (CT, CP, and CR) and overexpression groups (OT, OP, and OR) in the eyestalk at 4 h, 8 h, 12 h, 16 h, 20 h, and 24 h. b: Expression profile of *EcR* in the control (CE) and overexpression groups (OE) in the ovary at 12 h and 24 h. Data are presented as mean ± SD (*n* ≥ 3). Statistical analysis was performed using ANOVA followed by Duncan’s test (**a**). Student’s *t*-test was conducted (**b**). * indicates *p* < 0.05, ** indicates *p* < 0.01, and *** indicates *p* < 0.001.

After mRNA overexpression of *tim*, its transcript level was significantly higher than that in the control group (CT, CP and CR) at 4 h, 8 h, 12 h, 16 h, and 24 h (*p* < 0.001), with an overexpression efficiency above 70%, verifying the effectiveness of overexpression (Figure 9a). With the overexpression of the *tim* gene, the *cry1* mRNA was gradually decreased relative to the control group (CR) at 4 h, 12 h, and 20 h. Similarly, the *EcR* expression level was significantly reduced at 12 h and 24 h (*p* < 0.01) (Figure 9b).

## 4. Discussion

### 4.1. Ec-Tim Exhibits Characteristics of Tissue Specificity and Developmental Stage Dependence

Herein, we identified and characterized the *tim* gene from *E. carinicauda*. Bioinformatic prediction showed that the TIM protein in *E. carinicauda* contains a typical TIM domain at the N-terminus (residues 1229–1311). Phylogenetic analysis indicated that the TIM sequence exhibits the closest phylogenetic relationship with those of *P. monodon*. Transcript abundance of *Ec-tim* increased markedly during the Zoea II–III stages, suggesting potential functions in somatic growth and cell cycle progression [6,36]. Circadian clock genes are broadly expressed in the central nervous system and peripheral tissues to modulate multiple physiological processes [37,38,39]. In the whiteleg shrimp *Litopenaeus vannamei*, *tim* is highly expressed in the hepatopancreas and ventral nerve [40]. *Parhyale hawaiensis* adapts to various naturally occurring tides by regulating its circadian rhythm behavior through its circadian clock [41]. Different species modulate physiological processes to adapt to external environments through tissue-specific regulation of circadian clock gene expression. The eyestalk serves as a key neuroendocrine hub in crustaceans, producing neuropeptides and melatonin to control locomotor rhythms, retinal photoadaptation and ocular photosensitivity [23]. In adult prawns, *tim* shows high expression in the eyestalk. *Tim* displays inverse expression trends in eyestalk and ovary across gonadal maturation, peaking at G3 in the eyestalk and G4 in the ovary. This asynchronous expression indicates potential crosstalk of circadian signals between the two tissues. Predominant *Ec-tim* expression in the eyestalk implies its essential function in orchestrating central circadian rhythms [42]. *Tim* in ovary participates in local circadian regulation within the gonad. Taken together, *Ec-tim* exhibits tissue specificity and developmental stage-dependent patterns in *E. carinicauda*.

### 4.2. Effects of Different Light Colors and Photoperiods on the Expression of Ec-Tim

We further investigated how light spectra and photoperiods regulate *tim* expression in *E. carinicauda*. *Ec-tim* transcript abundance peaked significantly at 3 h after light exposure (*p* < 0.05), followed by regular oscillatory expression. This rapid transcriptional induction demonstrates that *Ec-tim* is highly light-sensitive, and its persistent rhythmic expression does not rely on continuous colored light stimulation. Photoperiod shifts modulated the phase, amplitude and overall transcript level of rhythmic *tim* expression. Importantly, *Ec-tim* oscillations persisted under constant darkness, confirming it is an endogenous core clock gene. Generally, core circadian clock genes display rhythmic expression entrainable by external light–dark cycles [43,44]. Compared with 0 L:24 D, 8 L:16 D, 12 L:12 D, and 24 L:0 D, the 16 L:8 D photoperiod yielded slightly higher *Ec-tim* expression amplitude, with the expression peak occurring at 18 h. This indicates that *tim* serves as a key circadian regulator primarily tuned by environmental photic signals [13,45]. Behaviorally, *E. carinicauda* exhibits higher nocturnal feeding and group activity; molting and spawning behaviors mostly take place at night or early dawn, and darkness exacerbates intraspecific cannibalism [46]. Under photoperiod conditions, *L. vannamei* also exhibits distinct nocturnal feeding rhythms and locomotor activity [47]. In *Euphausia superba*, seasonal clock gene rhythms are underpinned by a conserved 24 h basal circadian cycle [48]. In decapoda crabs, *Calcinus californiensis* and *Clibanarius albidigitus* avoid competitive exclusion through temporal niche differentiation, enabling stable coexistence of similar sympatric species [49]. Collectively, *Ec-tim* modulates multiple physiological processes in *E. carinicauda* via intracellular circadian rhythms, advancing our understanding of how core clock genes sustain circadian homeostasis and support adaptation to ambient light conditions.

### 4.3. Function of the Tim and Its Association with Ovarian Development in E. carinicauda

RNAi-mediated silencing of *Ec-tim* significantly downregulated *cry1* and *per* transcripts (*p* < 0.01). This result aligns with the canonical circadian mechanism, in which PER–TIM heterodimers translocate into the nucleus to repress clock cycle-mediated transcription [50,51]. Conversely, *Ec-tim* mRNA overexpression gradually inhibited *cry1* transcription, with significant suppression detected at 12 h and 20 h post-treatment (*p* < 0.001). These findings indicate that oscillatory *tim* expression triggers compensatory modulation of the endogenous circadian gene network to sustain systemic circadian rhythmicity [29].

To explore the potential association between *Ec-tim* and the molting hormone pathway, we further examined its regulation of *EcR*, a molecule promoting ovarian maturation in crustaceans [26]. Compared with the control group, *EcR* expression was significantly upregulated at 12 h and 24 h after *Ec-tim* knockdown (*p* < 0.01). We therefore hypothesize that *Ec-tim* may modulate reproductive processes through ecdysteroid signaling. However, as only transcriptional changes of *EcR* were detected here, further phenotypic investigations and analyses on ovarian development are required to verify this regulatory role. Notably, knockdown of *Ec-tim* leads to elevated *EcR* transcript abundance, implying that *Ec-tim* may repress *EcR* transcription. These distinct regulatory patterns imply that *tim* and *cry1* participate in a conserved transcription–translation feedback loop (TTFL) to fine-tune the crustacean reproductive axis. Circadian oscillation of these clock genes likely coordinates gonadal maturation via temporal gating of downstream reproductive gene expression [52]. Accumulating evidence indicates that core circadian clock genes are tightly associated with ovarian development. In *Eriocheir sinensis*, circadian rhythm-related genes are highly expressed in the eyestalks, brain and thoracic ganglion, and correlate with precocious sexual maturation [14]. In *Procambarus clarkii*, increased light intensity or extended photoperiod induces glutathione and vitellogenin secretion to promote oocyte development and maturation [53]. Developmental expression profiling suggests that *Ec-tim* may synchronize organismal behavioral rhythms with somatic growth, larval development and sequential gonadal maturation processes.

Our research has certain limitations. RNAi knockdown assays exhibited obvious temporal dependence. We only detected transcriptional changes of *EcR* after *Ec-tim* silencing; further molecular mechanisms are required to fully verify the regulatory axis linking *Ec-tim* and ovarian maturation. Another limitation of the present work lies in the lack of protein overexpression validation: generating custom antibodies for protein overexpression assays requires prolonged experimental cycles and substantial costs, restricting our current overexpression analysis to mRNA-level manipulation only.

## 5. Conclusions

In summary, this study identified and characterized *Ec-tim* in *E. carinicauda*. *Ec-tim* exhibited tissue-specific and developmental stage-dependent expression, and displayed endogenous circadian oscillations responsive to photic cues. RNAi assays revealed that knockdown of *Ec-tim* altered transcription of core clock genes (*per* and *cry1*) and significantly upregulated *EcR*. These results indicate that *Ec-tim* acts as a core circadian clock gene and may interact with the ecdysteroid signaling pathway. This work provides fundamental clues to understand the linkage between endogenous clock genes and reproductive regulation in crustaceans.

## Figures and Tables

**Table 1:** Primer sequences used in this study.

Primer Name	(5′-3′) Primer Sequence	Usage
*tim*-2170-F	AAAACTAATGGAAGGCAAAGGA	Core sequence amplification primers
*tim*-3661-R	TACGAATAAGCAGCACAAACAA	
*tim*-3647-F	TGCTGCTTATTCGTATGTACCGC	
*tim*-4790-R	CCATTTTCATCCCATTCATTGCTGT	
*tim*-3-4896	ATCGTCGGACCAGACATCCCAAAAC	3′RACE primers
*tim*-3-5332	ACAACCGCTCCAGTACATCATCCCC	
*tim*-3-5403	CAACAATCCAATCAGTTTTGCCACAGG	
*tim*-5-1343	TTGTCCTGGATTAGACGCCGAAGAA	5′RACE primers
*tim*-5-1448	GACTCCAAAATCCCTCTGTCTTCCTTCA	
*tim*-5-1552	CCAACTCGCTTATTATGCGGAACCCT	
*tim*-RNAi-A	GATCACTAATACGACTCACTATAGGGGCAGCATTTGTGGAATATATT	siRNA interference
*tim*-RNAi-B	AATATATTCCACAAATGCTGCCCCTATAGTGAGTCGTATTAGTGATC	
*tim*-RNAi-C	AAGCAGCATTTGTGGAATATACCCTATAGTGAGTCGTATTAGTGATC	
*tim*-RNAi-D	GATCACTAATACGACTCACTATAGGGTATATTCCACAAATGCTGCTT	
*tim*-O-F	TAATACGACTCACTATAAGGCAGGTCGACTCTATGTCTGATATTCCAAAATCTCGGA	Overexpression
*tim*-O-R	ACTATAGGGAGACCGGAATTCTTATGAATCTATTTCAAGAGCCTCACT	
*Ec-per*-F	CCTGAACCGCCACCTAATGT	qRT-PCR
*Ec-per*-R	GTCTGTGGGCAACTTCCGTA	
*Ec-tim*-F	CACAGGTTCCCTCAGAACCC	
*Ec-tim*-R	GGGATGATGTACTGGAGCGG	
*Ec-cry1*-F	AGAAGAGTGAACAGAGGGAAGC	
*Ec-cry1*-R	GGTAGCCCCAGTAAAGACGA	
*Ec*-18S-F	TATACGCTAGTGGAGCTGGAA	Internal reference
*Ec*-18S-R	GGGGAGGTAGTGACGAAAAAT	

## Data Availability

All data in this study are included in the article.
